# Vitamin C and MEK Inhibitor PD0325901 Synergistically Promote Oligodendrocytes Generation by Promoting DNA Demethylation

**DOI:** 10.3390/molecules29245939

**Published:** 2024-12-16

**Authors:** Xinyue Ren, Ying Yang, Min Wang, Qianting Yuan, Na Suo, Xin Xie

**Affiliations:** 1 State Key Laboratory of Drug Research, National Center for Drug Screening, Shanghai Institute of Materia Medica, Chinese Academy of Sciences, Shanghai 201203, China; s19-renxinyue@simm.ac.cn (X.R.); yangying1@shanghaitech.edu.cn (Y.Y.); mwang@simm.ac.cn (M.W.); qtyuan@simm.ac.cn (Q.Y.); 2 School of Pharmacy, University of Chinese Academy of Sciences, Beijing 100049, China; 3 School of Life Science and Technology, ShanghaiTech University, Shanghai 201210, China; 4 Shandong Laboratory of Yantai Drug Discovery, Bohai Rim Advanced Research Institute for Drug Discovery, Yantai 264117, China; 5 School of Pharmaceutical Science and Technology, Hangzhou Institute for Advanced Study, University of Chinese Academy of Sciences, Hangzhou 310024, China

**Keywords:** oligodendrocyte, oligodendrocyte progenitor cell, vitamin C, PD0325901, DNA demethylation, 5-hydroxymethylcytosine, TET enzymes

## Abstract

DNA methylation and demethylation are key epigenetic events that regulate gene expression and cell fate. DNA demethylation via oxidation of 5-methylcytosine (5mC) to 5-hydroxymethylcytosine (5hmC) is typically mediated by TET (ten-eleven translocation) enzymes. The 5hmC modification is considered an intermediate state of DNA demethylation; it is particularly prevalent in the brain and is believed to play a role in the development of many cell types in the brain. Our previous studies have identified that vitamin C (Vc) and MEK inhibitor PD0325901 could significantly promote OPC (oligodendrocyte progenitor cell)-to-OL (oligodendrocyte) differentiation. Here we discovered that Vc and PD0325901 may promote OPC-to-OL differentiation by inducing DNA demethylation via hydroxymethylation. Blocking 5hmC formation almost totally blocked Vc- and PD0325901-stimulated OPC-to-OL differentiation. In addition, TET1 is not involved in Vc,- and PD0325901-promoted OL generation. We also found a synergistic effect between the two compounds in inducing OL generation, suggesting the possibility of a combination therapy for demyelination diseases in the future.

## 1. Introduction

In the central nervous system, myelin sheath formed by oligodendrocytes (OLs) is a kind of electrical insulating membrane tightly wrapped around axons [1,2]. Myelin sheath is not only essential for signal transmission but also provides metabolic support to the axons, ensuring their long-term viability [3]. Developmentally, the generation of OLs from oligodendrocyte progenitor cells (OPCs) is a finely regulated, multi-step process influenced by various transcriptional, epigenetic, and environmental factors [4,5,6,7,8]. Platelet-derived growth factor receptor α (PDGFRα)- and chondroitin sulfate proteoglycan neuron-glia antigen 2 (NG2)-expressing OPCs first differentiate into immature OLs, which express antigens like O4 and 2′,3′-cyclic nucleotide-3′-phosphohydrolase (CNPase). Additionally, the expression of myelin antigens, such as myelin basic protein (MBP), proteolipid protein (PLP), myelin oligodendrocyte glycoprotein (MOG), and myelin-associated glycoprotein (MAG), is the sign of OL maturation. Mature OLs possess the capacity to form myelin.

Dysfunctional or structural damage to OLs leads to myelin loss. The loss of myelin, also termed demyelination, disrupts signal transmission in neurons and triggers axonal damage, eventually leading to neurological deficits and disability [9,10,11,12]. Myelin regeneration, or remyelination, is a natural repair process that can occur in response to demyelination [13,14,15]. During remyelination, widely distributed adult OPCs are activated and differentiate into mature OLs, which generate new myelin to wrap around the exposed axons. Remyelination not only restores nerve conduction but also provides metabolic support to protect axons from degeneration [16]. The efficacy of remyelination, however, is variable. Remyelinated axons may display thinner myelin sheaths than their original counterparts. In progressive stages of diseases like multiple sclerosis (MS), remyelination becomes less efficient, partly due to the inability of OPCs to differentiate into mature OLs. Over the past 20 years, plenty of researchers have dedicated their research to identify active molecules that promote OL differentiation and enhance myelin regeneration [17]. Based on these findings, current clinical trials are exploring several therapeutic targets for remyelination. Unfortunately, to date, drug treatment for remyelination is still an unmet need [18,19].

Previously, we have discovered that vitamin C (Vc) dramatically promotes the differentiation of OPCs into OLs in vitro as well as myelin regeneration in vivo [20]. However, the mechanism of the benefit effect of Vc on OPC-to-OL differentiation was still not clear. Apart from antioxidant activity, Vc regulates the activity of TET (ten-eleven translocation) enzymes, which mediate the oxidation of 5-methylcytosine (5mC) to 5-hydroxymethylcytosine (5hmC) and play a critical role in DNA demethylation [21]. We have proven that the antioxidant effect of Vc is not involved in its promotion of OPC-to-OL differentiation, and we wondered whether the epigenetic modulation effect of Vc is involved in this process.

We have also reported that inhibition of the MAPK/ERK pathway promotes OL generation [22,23]. Interestingly, several studies have indicated that MEK inhibitors may also influence DNA methylation [24,25]. The combination of a MEK inhibitor and a GSK3 inhibitor has been reported to maintain a ground state of mouse embryonic stem cells (ESCs) by downregulating global DNA methylation [24]. In this process, the MEK inhibitor increases the JMJD2C protein level but decreases DNMT3 expression to elevate the 5hmC level [24]. MEK inhibitor PD0325901 has also been reported to repress DNMT3b and its cofactor DNMT3l at the protein level, resulting in a moderately reduced level of global DNA methylation [25].

These studies suggest that Vc and MEK inhibitors may affect the epigenetic modifications of DNA by inducing DNA demethylation via hydroxymethylation. In this study, we sought to investigate the involvement of DNA demethylation in Vc- and PD0325901-mediated OPC-to-OL differentiation and the potential synergistic effect between the two, which might be used in combination in future applications.

## 2. Results

### 2.1. Involvement of Active DNA Demethylation in As-2P-Induced OPC-to-OL Differentiation

**Figure 1:**
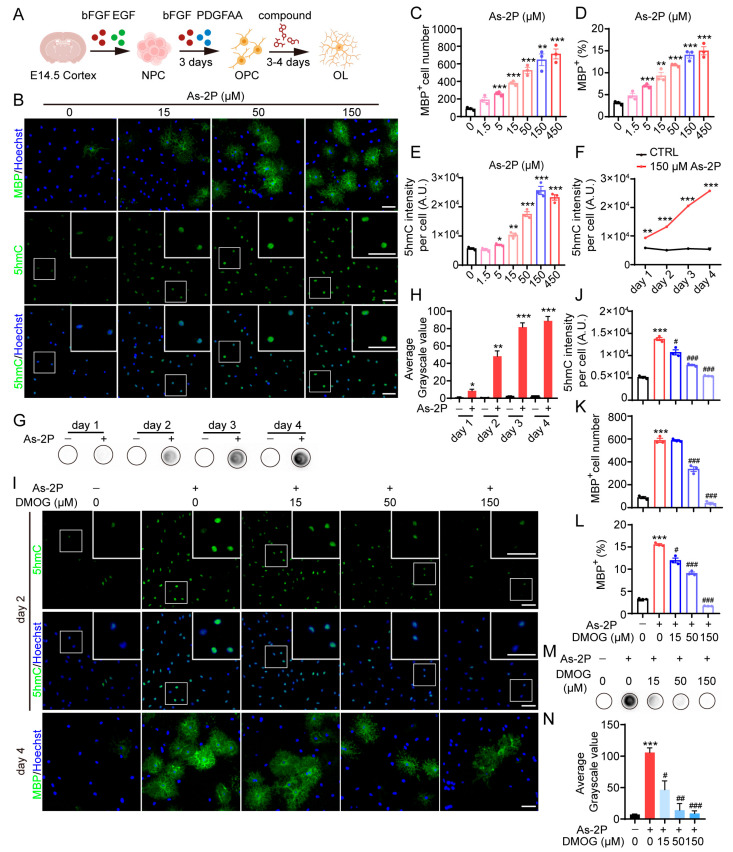
Blocking TET-mediated DNA demethylation blocks As-2P-induced OPC-to-OL differentiation. (**A**) An illustration of NPC to OPC and then to OL differentiation. Cortical NPCs were isolated from E14.5 mouse embryos and expanded in vitro as neurospheres in the presence of bFGF and EGF. OPCs were induced by treating NPCs with bFGF and PDGF-AA for 3 days. OPCs were further differentiated into MBP^+^ OLs by removing growth factors, in the presence or absence of the test compounds. (**B**) Representative images of mouse OLs induced from NPC-derived OPCs with various concentrations of As-2P for 4 days. OLs were stained with antibodies against MBP (green) or 5hmC (green). Nuclei were stained with Hoechst 33342 (blue). Scale bars, 100 μm. (**C**–**E**) Statistical analysis of the number and the percentage of MBP^+^ cells (**C**,**D**) and the intensity of 5hmC (**E**) in (**B**). (**F**) Statistical analysis of 5hmC intensity during OPC-to-OL differentiation with 150 μM As-2P. (**G**) Dot blot analysis of 5hmC levels during OPC-to-OL differentiation with or without 150 μM As-2P. For each dot, 40 ng genomic DNA was loaded. (**H**) Statistical analysis of 5hmC level in (**G**). (**I**) Representative images of mouse OLs induced with As-2P (150 μM) in combination with various concentrations of DMOG. The 5hmC (green) and MBP (green) were detected with immunofluorescent staining at days 2 and 4. Nuclei were stained with Hoechst 33342 (blue). Scale bars, 100 μm. (**J**–**L**) Statistical analysis of the intensity of 5hmC (**J**) and the number and percentage of MBP^+^ cells (**K**,**L**) in (**I**). (**M**) Dot blot analysis of 5hmC levels during OPC-to-OL differentiation in the presence of 150 μM As-2P and various concentrations of DMOG. For each dot, 40 ng genomic DNA was loaded. (**N**) Statistical analysis of 5hmC levels in (**M**). Data are presented as means ± SEM (*n* = 3). * *p* < 0.05, ** *p* < 0.01, *** *p* < 0.001 versus CTRL group, ^#^ *p* < 0.05, ^##^ *p* < 0.01, ^###^ *p* < 0.001 versus As-2P group (Student’s *t*-test).

As previously reported, we have established an in vitro NPC to OPC and then to OL differentiation system (Figure 1A). With this system, we have reported several small molecules and several GPCRs that regulate OPC to OL differentiation [20,22,23,26,27,28]. Among them, As-2P, a stable form of Vc, was highly effective in inducing OL generation in vitro and remyelination in vivo [20]. Although we have proven that As-2P′s effect is independent of its antioxidant property, the exact mechanism remains elusive.

Vc has been reported to regulate DNA demethylation via activation of TET dioxygenases, which catalyze the transformation of 5mC to 5hmC, a critical step in active DNA demethylation [29,30,31,32]. We wondered whether this process is also involved in Vc-stimulated OPC-to-OL differentiation. To verify this, we detected both MBP and 5hmC in the differentiation process. Indeed, As-2P dose-dependently promoted the generation of MBP^+^ OLs from NPC-derived OPCs (Figure 1B–D). Meanwhile, a dose-dependent elevation of 5hmC was also observed (Figure 1B,E). During the whole process of OPC-to-OL differentiation, As-2P induced a gradual accumulation of 5hmC over time (Figure 1F). Dot blot analysis of 5hmC also confirmed the result (Figure 1G,H).

DMOG is a non-selective TET dioxygenase inhibitor that has been reported to simultaneously inhibit the activity and expression of TET1–3 [33,34]. Adding DMOG to the differentiation system significantly reduced As-2P-elevated 5hmC levels (Figure 1I,J,M,N). At a high dose, DMOG almost completely blocked As-2P-induced generation of MBP^+^ OLs (Figure 1J–L). These results suggest that active DNA demethylation mediated by TETs plays a critical role in As-2P-stimulated OPC-to-OL generation.

### 2.2. TETs-Mediated DNA Demethylation Is Also Involved in PD0325901-Induced OPC-to-OL Differentiation

**Figure 2:**
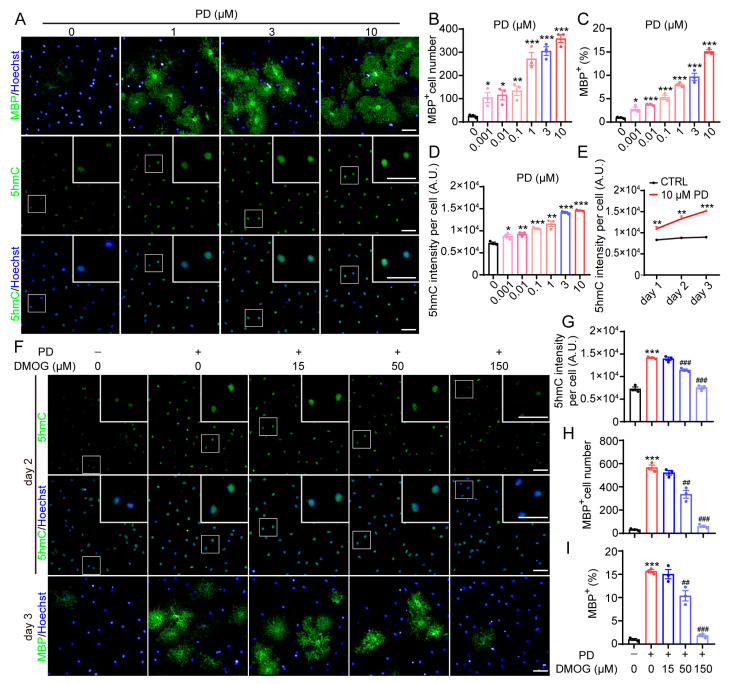
Blocking TET-mediated DNA demethylation blocks PD0325901-induced OPC-to-OL differentiation. (**A**) Representative images of mouse OLs induced with various concentrations of PD0325901 for 3 days. OLs were stained with antibodies against MBP (green) or 5hmC (green). Nuclei were stained with Hoechst 33342 (blue). Scale bars, 100 μm. (**B**–**D**) Statistical analysis of the number and the percentage of MBP^+^ (**B**,**C**) cells and the intensity of 5hmC (**D**) in (**A**). (**E**) Statistical analysis of the intensity of 5hmC during OPC-to-OL differentiation with 10 μM PD0325901 for 1–3 days. (**F**) Representative images of mouse OLs induced with PD0325901 (10 μM) in combination with various concentrations of DMOG. The 5hmC (green) and MBP (green) were detected at day 2 and 3. Nuclei were stained with Hoechst 33342 (blue). Scale bars, 100 μm. (**G**–**I**) Statistical analysis of the intensity of 5hmC (**G**) and the number and percentage of MBP^+^ cells (**H**,**I**) in (**F**). Data are presented as means ± SEM (*n* = 3). * *p* < 0.05, ** *p* < 0.01, *** *p* < 0.001 versus CTRL group, ^##^ *p* < 0.01, ^###^ *p* < 0.001 versus PD0325901 group (Student’s *t*-test).

We have also reported that MAPK/ERK inhibitor PD0325901 could promote OPC-to-OL differentiation by blocking the ERK signaling [22]. We wondered whether the TETs-mediated DNA demethylation is also involved in PD0325901-induced OPC-to-OL differentiation. PD0325901 could dramatically promote the differentiation of OPCs to OLs in a dose-dependent manner (Figure 2A–C), and a dose- and time-dependent elevation of 5hmC was also observed after PD0325901 treatment (Figure 2A,D,E). Similar to the As-2P-induced OL differentiation, the TET inhibitor DMOG not only reduced PD0325901-elevated 5hmC content but also completely blocked PD0325901-stimulated OPC-to-OL differentiation at a high dose (Figure 2F–I). These results imply that active DNA demethylation is a critical step regulating As-2P- and PD0325901-mediated OL differentiation, no matter the differences in upstream pathways.

### 2.3. Tet1 Is Not Involved in As-2P- or PD0325901-Mediated OPC-to-OL Differentiation

**Figure 3:**
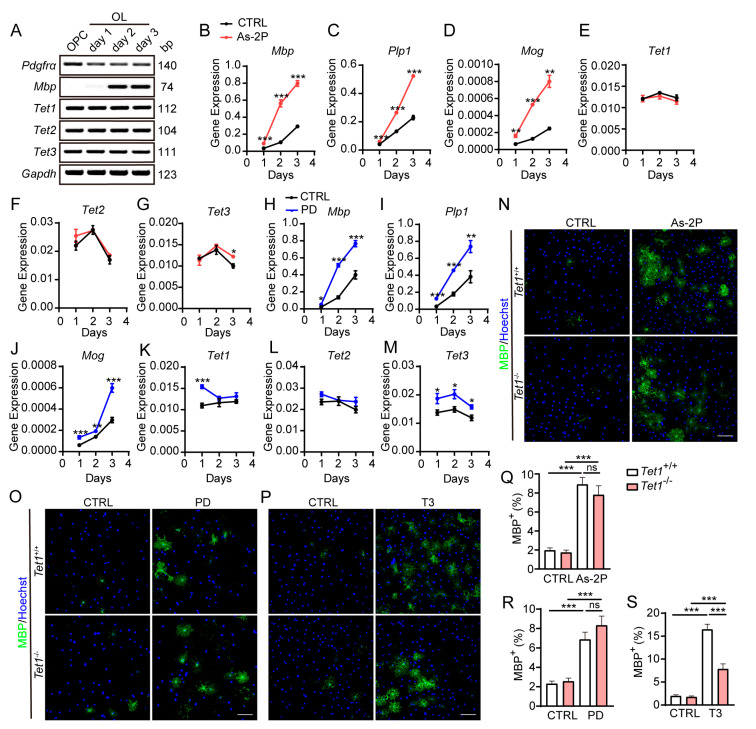
*Tet1* deficiency does not affect OPC-to-OL differentiation induced either by As-2P or PD0325901. (**A**) The expression levels of *Tet1*, *Tet2*, and *Tet3* were detected by RT-PCR during OPC to OL differentiation. *Gapdh* was used as a loading control. *Pdpfra* and *Mbp* were markers for OPCs and mature OLs, respectively. (**B**–**M**) The expression levels of *Mbp*, *Plp1*, *Mog*, *Tet1*, *Tet2*, and *Tet3* were detected by qPCR at various time points during OPC to OL differentiation with 150 μM As-2P (**B**–**G**) or 10 μM PD0325901 (**H**–**M**). Data are normalized to *Gapdh*, and presented as means ± SEM (*n* = 3). * *p* < 0.05, ** *p* < 0.01, *** *p* < 0.001 versus CTRL group (Student’s *t*-test). (**N**–**P**) Representative images of OLs induced from WT or Tet1 KO primary mouse OPCs in the presence of 150 μM As-2P (**N**), 10 μM PD0325901 (**O**), or 100 nM T3 (**P**) for 4 days. MBP (green) were detected with antibodies, and nuclei were stained with Hoechst 33342 (blue). Scale bars, 100 μm. (**Q**–**S**) Statistical analysis of the percentage of MBP^+^ cells induced from WT or *Tet1* KO primary mouse OPCs with 150 μM As-2P (**Q**), 10 μM PD0325901 (**R**), and 100 nM T3 (**S**). Data are presented as means ± SEM (*n* = 3). *** *p* < 0.001 (Student’s *t*-test); ns, no significance.

Active DNA demethylation via hydroxy methylation is mediated by three TET dioxygenases. We attempted to determine the subtype of TET dioxygenases that might be involved in As-2P- and PD0325901-induced OPC-to-OL differentiation. During OPC differentiation, gradual downregulation of *Pdgfra*, a marker of OPCs, and dramatic upregulation of *Mbp*, a marker of mature OLs, could be observed. All 3 *Tet* genes, *Tet1*, *Tet2*, and *Tet3*, were steadily expressed in OPC/OL lineage cells (Figure 3A). As-2P treatment dramatically increased the level of myelin-related genes, including *Mbp*, *Plp1*, and *Mog*, without affecting the levels of *Tet1*, *Tet2*, and *Tet3* (Figure 3B–G). PD0325901 induced similar levels of myelin genes as As-2P and slightly increased *Tet1* and *Tet3* expression (Figure 3H–M). Although all three TETs are responsible for the conversion of 5mC to 5hmC, only TET1 has been reported to be involved in OL development and myelination [35,36]. Hence, the role of TET1 in As-2P- and PD0325901-induced OPC-to-OL differentiation was studied. Mouse primary OPCs were isolated from *Tet1*^+/+^ and *Tet1*^−/−^ mice and induced to differentiate into MBP^+^ OLs for 4 days with 150 μM As-2P, 10 μM PD0325901, or 100 nM T3. It was interesting to observe that *Tet1*^−/−^ did not affect the differentiation efficiency induced by As-2P or PD0325901 (Figure 3N,O,Q,R) but significantly inhibited T3-induced OLs generation (Figure 3P,S). These results imply that the beneficial effects of As-2P and PD0325901 on OPC-to-OL differentiation are not mediated by TET1, although it does play a role in OL differentiation.

### 2.4. As-2P and PD0325901 Display Synergistic Effect in Promoting OPC-to-OL Differentiation

**Figure 4:**
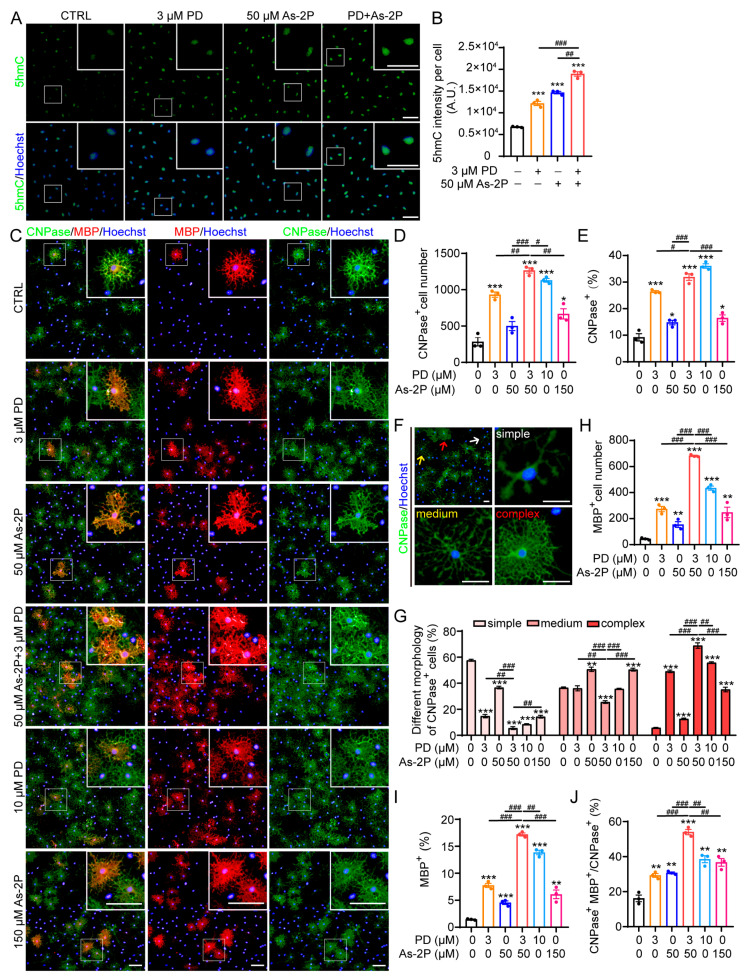
As-2P and PD0325901 display a synergistic effect in inducing DNA demethylation and OPC-to-OL differentiation. (**A**) The effect of As-2P, PD0325901, and the combination of both in inducing DNA demethylation during OL differentiation. NPC-derived OPCs were treated with indicated compound (s) in differentiation medium for 2 days, and 5hmC (green) was detected with immunofluorescent staining. Nuclei were stained with Hoechst 33342 (blue). Scale bars, 50 μm. (**B**) Statistical analysis of the 5hmC intensity in (**A**). (**C**) Representative images of mouse OLs induced with PD0325901 (3 or 10 μM), As-2P (50 or 150 μM), or the combination of PD0325901 (3 μM) and As-2P (50 μM) for 2 days. Cells were stained with antibodies against CNPase (green) and MBP (red). Nuclei were stained with Hoechst 33342 (blue). Scale bars, 100 μm. (**D**,**E**) Statistical analysis of the number and percentage of CNPase^+^ cells in (**C**). (**F**,**G**) Morphological classification ((**F**), simple: multiple process outgrowth; medium: extensive process outgrowth and branching; complex: terminal membrane expansion) and statistical analysis (**G**) of CNPase^+^ cells based on morphology in (**C**). Scale bars in (**F**), 50 μm. (**H**–**J**) The number and the percentage of MBP^+^ cells (**H**,**I**) and the percentage of MBP^+^CNPase^+^ cells within CNPase^+^ cells (**J**) in (**C**). Data are presented as means ± SEM (*n* = 3). * *p* < 0.05, ** *p* < 0.01, *** *p* < 0.001 versus CTRL group, ^#^ *p* < 0.05, ^##^ *p* < 0.01, ^###^ *p* < 0.001 versus PD or As-2P group (Student’s *t*-test).

For possible in vivo or clinical application, lower concentrations of drugs typically translate to fewer side effects. The reduced efficacy due to low concentration could be rescued by a drug combination. We wondered whether As-2P and PD0325901 could be combined at low concentrations to achieve better efficacy than a single compound at higher concentrations. The combination of 3 μM PD0325901 and 50 μM As-2P resulted in a greater increase in DNA 5hmC levels compared to 3 μM PD0325901 or 50 μM As-2P alone (Figure 4A,B). Moreover, the combination of 3 μM PD0325901 and 50 μM As-2P was most effective in inducing OPCs-to-OL differentiation, even better than 10 μM PD0325901 or 150 μM As-2P alone (Figure 4C–J).

During the process of OPCs differentiation, O4, CNPase, MBP, PLP, MOG, and MAG are gradually expressed in sequence. We first analyzed the number and proportion of CNPase^+^ immature OLs induced by different treatments and found that combining 3 μM PD0325901 and 50 μM As-2P induced the largest number of immature OLs (Figure 4C–E). The maturation of OLs is a process of morphological change characterized by complex process extension and membrane formation. Within the CNPase^+^ immature OLs, cells at different maturation stages could be classified into ‘simple’, ‘medium’, or ‘complex’ according to their morphology (Figure 4F). Cells treated with the combination of 50 μM As-2P and 3 μM PD0325901 yielded the highest percentage of CNPase^+^ OLs with complex morphology and the lowest percentage of cells with simple morphology, indicating that the combination significantly accelerated the differentiation and maturation process (Figure 4G). Indeed, when evaluating with mature OL marker MBP, the combination was the most effective in inducing the generation of MBP^+^ mature OLs, even more effective than As-2P or PD0325901 at higher concentrations (Figure 4C,H–J). Taken together, As-2P and PD0325901 could synergistically promote OL generation by promoting DNA demethylation.

## 3. Discussion

DNA methylation and demethylation are key epigenetic events that regulate gene expression, development, and disease [30,37]. DNA methylation involves the addition of a methyl group to the fifth carbon of cytosine, primarily in CpG dinucleotides [38,39]. It is closely associated with transcriptional repression. Methylation patterns are heritable through cell division, and their establishment and maintenance are mediated by DNMTs [40]. Hydroxymethylation, on the other hand, is a more dynamic modification where 5mC is converted to 5hmC by TET enzymes. This modification is particularly prevalent in the brain and is believed to play a role in neural development and plasticity, as well as OL development [41,42,43]. Hydroxymethylation is considered an intermediate step in active DNA demethylation, potentially reversing the gene-silencing effects of methylation. TET enzymes oxidize 5mC to 5hmC, facilitating the removal of methyl groups, which is essential for processes like embryonic development and cellular differentiation [44,45]. Both modifications are crucial for normal development, and their dysregulation is linked to various diseases, including cancer and neurodevelopmental disorders.

Here we discovered that 5hmC formation was critically involved in both Vc- and PD0325901-stimulated OPC-to-OL differentiation, since blocking 5hmC formation by DMOG almost completely blocked the differentiation. Vc did not affect the expression of *Tets* in OPCs, consistent with previous observations in other cell types [21,46]. Vc is likely to regulate 5hmC level in OPCs by modulating TETs activity, similar to what has been observed in other cells, including mouse embryonic fibroblasts, bladder cancer cells, and astrocytes [31,47,48]. On the other hand, PD0325901 may upregulate *Tets*, especially *Tet3*, during OPC-to-OL differentiation.

TET enzymes have been reported to play important roles in OL formation, myelination, and remyelination. TET1-mediated DNA demethylation is essential for efficient myelin repair in the CNS; specific age-related declines in remyelination efficiency have been reported in TET1-deficient models [35,36]. *Tet1* ablation in OPCs has also been shown to impair myelin development in mice [35]. TET2 and TET3, while also involved in OL differentiation, display unique temporal and spatial expression patterns during OL development [43]. Although all three TET proteins are necessary for proper OL maturation [43], TET1′s role in regulating key epigenetic processes appears to be more pronounced during both developmental myelination and adult remyelination [35,36]. TET2 and TET3 do not show significant effects in myelination when knocked out, suggesting a more supportive role compared to TET1 [35,36]. However, we found that knocking out *Tet1* did not affect vitamin C- or PD0325901-mediated OPC differentiation, although it did reduce thyroid hormone T3-mediated differentiation. TET1, TET2, and TET3 are all expressed in OL lineage cells [43], and Vc could activate all three of these enzymes. In the presence of Vc, activation of TET2 and TET3 may compensate for the loss of TET1. In the case of PD0325901, its upregulation of TET3 may also compensate for the loss of TET1. These require further evaluation through double or triple knockouts of *Tets*.

Another interesting finding here is that the combination of Vc and PD0325901 showed a synergistic effect in inducing 5hmC and promoting OL generation, indicating that the combination of these compounds at relatively low concentrations could reach a better effect than a single compound at higher concentrations. This is important for in vivo or clinical application, as lower concentrations of drugs typically translate to fewer side effects. Vc could reach a 2–10 mM level in the human brain [49]. However, PD0325901 could only reach a low µM level in human blood when given at 15 mg/kg twice daily in a Phase 1 trial [50]. Considering the low brain/plasma ratio of PD0325901 [51,52,53], it is challenging to deliver an effective dose of PD0325901 to the CNS to promote myelin regeneration in humans. A combination of PD0325901 and Vc might be more practical in possible clinical studies.

Whether PD0325901 directly modulates the activity of TETs warrants further investigation. The activity of TET enzymes is regulated by multiple pathways, ensuring that their role in DNA demethylation is precisely controlled. These pathways include interactions with cofactors, post-translational modifications, and metabolic regulation [54]. One key mechanism involves the dependence of TET enzymes on cofactors such as Fe^2+^ and α-KG. Vc helps to maintain Fe^2+^ in its reduced state, thereby boosting TET enzymes’ activity [55]. Additionally, TET enzymes undergo various post-translational modifications, including phosphorylation, acetylation, and ubiquitination, which influence their stability and activity [54]. Phosphorylation by kinases such as AMPK and CDK5 alters TET2 and TET3 activity, respectively, promoting their roles in DNA demethylation and cell differentiation [56]. O-linked glycosylation of TET enzymes affects their interactions with partners, modulating their stability, localization, and function [57,58]. Metabolites like succinate and fumarate can inhibit TET activity by competing for the α-KG binding site, effectively reducing TET-mediated DNA demethylation [59]. The activity of TET enzymes is also modulated by various partner proteins [54]. Transcription factors such as NANOG and STAT3 interact with TET1 and TET2, guiding them to specific DNA regions to influence gene expression [60,61]. The MAPK pathway involves many kinases, and it is likely that MEK inhibitor PD0325901 may directly or indirectly modulate TET activity through some of the above pathways during OL generation.

In conclusion, we discovered that Vc and MEK inhibitor PD0325901 may promote OPC-to-OL differentiation by inducing DNA demethylation via hydroxymethylation. We also found a synergistic effect between the two compounds in inducing OL generation, suggesting the possibility of a combination therapy for demyelination diseases in the future.

## 4. Materials and Methods

### 4.1. Reagents

Laminin, poly-ornithine, paraformaldehyde (PFA), Hoechst 33342, 2-Phospho-l-ascorbic acid trisodium salt, thyroid hormone (T3), and BSA were purchased from Sigma Aldrich (St. Louis, MO, USA). EGF, bFGF, and PDGF-AA were purchased from Peprotech (Cranbury, NJ, USA). DMOG and PD0325901 were purchased from Targetmol (Shanghai, China).

### 4.2. NPC-Derived OPCs Differentiation

Neural progenitor cells (NPCs) were isolated and purified from dissected cerebral cortexes of E14.5 mouse embryos by suspension culture. NPCs were expanded as neural spheres in NPC medium (DMEM/F12 (Gibco, Grand Island, NY, USA) supplemented with 20 ng/mL EGF, 20 ng/mL bFGF, 2% B27 (Invitrogen, Carlsbad, CA, USA), 100 units/mL penicillin (Invitrogen), and 100 μg/mL streptomycin (Invitrogen)) and passaged every two days. NPCs from passages 3–5 were used to induce differentiation. To generate OPCs, neural spheres were dissociated into single cells with accutase (Millipore, Bedford, MA, USA, SF006) and seeded onto poly-ornithine (25 μg/mL) plus laminin (1 μg/mL)-coated plates in OPC medium (DMEM/F12 supplemented with 10 ng/mL bFGF, 10 ng/mL PDGF-AA, 2% B27, 100 units/mL penicillin, and 100 μg/mL streptomycin). After three days, PDGF-AA and bFGF were removed, and testing compounds or 0.1% DMSO (control) were added for another 3–4 days to induce the differentiation of OPCs into MBP^+^ OLs.

### 4.3. Tet1 Knockout Mouse

*Tet1*^+/−^ mice (deletion of the exon 4 of the *Tet1* gene) were purchased from GemPharmatech Co., Ltd. (Nanjing, China). *Tet1*^+/−^ mice were obtained by mating *Tet1*^+/−^ mice of different genders. Genotypes of the progeny were determined by PCR of the tail DNA according to the manufacturer’s instructions. For OPC isolation, P5 neonatal *Tet1*^+/−^ mice and their littermates were used. All animal experiments were conducted in accordance with the international guidelines for the care and use of laboratory animals and approved by the Animal Ethics Committee of Shanghai Institute of Materia Medica.

### 4.4. Primary OPCs Isolation and Differentiation

To isolate primary OPCs from mice, cortices of newborn mice (P5) were collected in cold Hank’s balanced salt solution (HBSS) and dissociated using a neural tissue dissociation kit (Miltenyi, Bergesch Gladbach, Germany, 130-092-628) according to the manufacturer’s instructions. Cortical cells were then sorted by anti-AN2 (anti-NG2) microbeads (Miltenyi, 130-097-170) to isolate NG2^+^ OPCs. Primary OPCs were seeded onto plates coated with poly-ornithine (25 μg/mL) plus laminin (1 μg/mL) in OPC medium. Twenty-four hours later, OPCs were induced to differentiate into MBP^+^ OLs in the absence of bFGF and PDGFAA but with testing compounds or 0.1% DMSO as the control for 3–4 days.

### 4.5. Immunofluorescence Staining

Cells were fixed with 4% PFA in phosphate-buffered saline (PBS) for 15 min and blocked in PBS containing 2.5% BSA and 0.3% Triton for 30 min at room temperature. Then cells were incubated with the relevant primary antibody (anti-MBP (Millipore, Bedford, USA, MAB386, 1:500) or anti-CNPase (Millipore, MAB326, 1:500)) at 4 °C overnight. After thoroughly washing, cells were stained with the appropriate secondary antibody conjugated to Alexa Fluor 488 or Alexa Fluor 555 for 1 h at room temperature (Invitrogen, A-11001, A-21434, 1:1000). Nuclei were stained with Hoechst 33342 (5 μg/mL).

For DNA 5hmC staining, cells were fixed with 4% PFA in PBS for 15 min and permeabilized in PBS containing 0.3% Triton for 45 min at room temperature. Chromatin was denatured with 2 N HCl for 30 min and washed twice with 100 mM Tris-HCl pH 8.0 for 5 min at room temperature. Blocking was performed in 2.5% BSA for 1 h. Then cells were incubated with anti-5hmC (Active Motif, Carlsbad, CA, USA, #39769, 1:1000) at 4 °C overnight. After thoroughly washing, cells were stained with goat anti-rabbit IgG (H + L) cross-adsorbed secondary antibody (Invitrogen, A-11008, 1:1000) for 1 h at room temperature. Nuclei were stained with Hoechst 33342 (5 μg/mL). Images were taken by an Opera Phenix High Content Screening System (Revvity, Waltham, MA, USA), and image analysis was performed by Image J (V1.8.0).

### 4.6. Reverse Transcription and PCR

Total RNA was subjected to reverse transcription with the PrimeScript RT Reagent Kit (Takara Bio, Kyoto, Japan, RR037A). PCR was conducted in an ABI Veriti FAST gradient PCR instrument (Thermo, Waltham, MA, USA) using 2× Taq Master Mix (Dye Plus) (Vazyme, Nanjing, China). To detect *Tets* in OPCs and OLs, PCR was performed using diluted reverse transcription products, 30 cycles were used for *Tet1*, *Tet2*, and *Tet3* transcripts amplification, and 27 cycles were used for *Pdgfrα*, *Mbp*, and *Gapdh* transcripts amplification. All the products of RT-PCR were analyzed by 2% agarose gel electrophoresis. Realtime PCR was performed using the FastStart Universal Probe Master Mix (Bimake, Houston, TX, USA, B21702) and a Stratagene Mx3000P thermal cycler (Palo Alto, CA, USA). The sequences of the primer pairs are as follows: *Tet1*, sense (5′-ACACAGTGGTGCTAATGCAG-3′), and antisense (5′-AGCATGAACGGGAGAATCGG-3′); *Tet2*, sense (5′-AGAGAAGACAATCGAGAAGTCGG-3′), and antisense (5′-CCTTCCGTACTCCCAAACTCAT-3′); *Tet3*, sense (5′-TGCGATTGTGTCGAACAAATAGT-3′), and antisense (5′-TCCATACCGATCCTCCATGAG-3′); *Mbp*, sense (5′-GGCGGTGACAGACTCCAAG-3′), and antisense (5′-GAAGCTCGTCGGACTCTGAG-3′); *Plp1*, sense (5′-TGAGCGCAACGGTAACAGG-3′), and antisense (5′-TTCCCAAACAATGACACACCC-3′); *Mog*, sense (5′-AGCTGCTTCCTCTCCCTTCTC-3′), and antisense (5′-ACTAAAGCCCGGATGGGATAC-3′); *Pdgfrα*, sense (5′-TCCATGCTAGACTCAGAAGTCA-3′), and antisense (5′-TCCCGGTGGACACAATTTTTC-3′); *Gapdh*, sense (5′-AGGTCGGTGTGAACGGATTTG-3′), and antisense (5′-TGTAGACCATGTAGTTGAGGTCA-3′).

### 4.7. Dot Blot

The genomic DNA of cells was collected and extracted using the Wizard Genomic DNA Purification Kit (Promega, Madison, WI, USA). After denaturing with 2.5 M NaOH, DNA samples were slowly dropped onto the nitrocellulose membrane. Membranes were first dried at room temperature for 15 min, and then DNA was fixed on the nitrocellulose membrane by heating at 80 °C for 30 min. After that, membranes were incubated in TBST containing 5% BSA at room temperature for 1 h. Then it was incubated in TBST containing 5% BSA with anti-5hmC antibody (Active motif, Carlsbad, USA, #39769, 1:10,000) at 4 °C overnight. After thorough washing, the membranes were incubated in TBST containing the secondary antibody (anti-rabbit IgG HRP, CST, Danvers, MA, USA, 7074, 1:8000) at room temperature for 2 h. Finally, blots were visualized using an Omni-ECL™ Femto Light Chemiluminescence Kit (Epizyme, Shanghai, China, SQ201).

### 4.8. Statistical Analysis

Data were analyzed with GraphPad Prism software (V 8.0.0). For comparison between two groups, statistical evaluation was carried with two-tailed Student’s *t*-test. For all statistical tests, *p* values < 0.05 were considered statistically significant. All error bars show the standard error of the mean (SEM).

## Data Availability

The datasets used and/or analyzed during the current study are available from the corresponding author on reasonable request.
